# The Pharmacological Activity of the Wenjing Decoction in Recurrent Spontaneous Abortion

**DOI:** 10.1155/2021/8861394

**Published:** 2021-04-13

**Authors:** Linhui Cao, Hui Chen, Yanxia Huang, Liudan Chen, Mengru Kang, Junxiong Liang

**Affiliations:** ^1^Department of Traditional Chinese Medicine, Sun Yat-sen Memorial Hospital, SunYat-sen University, Guangzhou 510120, Guangdong, China; ^2^Department of Obstetrics and Gynecology, Sun Yat-sen Memorial Hospital, Sun Yat-sen University, Guangzhou 510120, Guangdong, China

## Abstract

**Background:**

Recurrent spontaneous abortion (RSA) is intractable infertility and can be ameliorated with the use of traditional Chinese medicine preparation, the Wenjing decoction. This study aimed to identify the therapeutic mechanism of Wenjing decoction on specific target proteins involved in RSA.

**Methods:**

Wenjing decoction contains Wuzhuyu, Danggui, Chuanxiong, Guizhi, Shengjiang, Banxia, Gancao, Ejiao, Mudanpi, Chishao, Dangshen, and Maidong. Using TCMSP and BATMAN databases, we queried for active ingredients and predicted their target proteins by BATMAN. Using the edgeR package, we analyzed the differentially expressed genes (DEGs) in the GSE121950 database between control samples and RSA (*n* = 3). The interaction between DEGs and the predicted target proteins was identified by the Venn diagram. Using the Cytoscape software and clusterProfiler package, enrichment analysis was conducted for the intersected target proteins. Additionally, the protein-protein interaction (PPI) network and pharmacological network were generated using the Cytoscape software.

**Results:**

In total, 31, 2, 7, 7, 5, 13, 93, 11, 29, and 21 active ingredients were identified from Wuzhuyu, Danggui, Chuanxiong, Guizhi, Shengjiang, Banxia, Gancao, Mudanpi, Chishao, and Dangshen, respectively. Additionally, 100 intersected target proteins were revealed by the Venn diagram. Moreover, 98 functional terms and 24 pathways (including C-type lectin receptor signaling pathway, chemokine signaling pathway, leukocyte transendothelial migration, fluid shear stress, and atherosclerosis, and AGE-RAGE signaling pathway in diabetic complications) were enriched. In the PPI network, 10 proteins involved in these five pathways were identified, namely, TNF-*α* (tumor necrosis factor-*α*), IL-10 (interleukin-10), TLR4 (Toll-like receptor 4), JUN (Jun proto-oncogene), IL-1B (interleukin-1-beta), CYBB (cytochrome b558 heavy chain gene), PTGS2 (prostaglandin-endoperoxide synthase 2), APOE (apolipoprotein E), SPI1 (salmonella pathogenicity island 1), and MPO (myeloperoxidase) which showed higher degrees.

**Conclusion:**

The abovementioned genes and pathways might be involved in the pharmacological activity of Wenjing decoction in RSA.

## 1. Introduction

Abortion involves pregnancy termination before 28 weeks, with fetal weight below 1000 g [1]. Abortion can be classified as spontaneous or induced abortion, and recurrent spontaneous abortion (RSA) involves two or more consecutive spontaneous abortions [2]. Abortion manifests as postmenopausal vaginal bleeding and abdominal pain, although some women do not exhibit any clinical symptoms [3]. Nearly 50% of women with RSA have identifiable etiologies, such as chromosomal abnormalities, cervical insufficiency, immune dysfunction, maternal endocrine abnormalities, maternal reproductive tract abnormalities, reproductive tract infection, and thrombotic susceptibility [4, 5]. RSA is intractable infertility clinically and poses a global therapeutic challenge, accounting for 1–5% of total pregnancies [6]. Therefore, it is vital to understand RSA pathogenesis to improve therapy.

Some traditional Chinese medicines (TCMs) have shown promise in women with recurrent miscarriage by improving embryo development and live birth rate [7]. The TCM Wenjing decoction improves blood circulation and eliminates stasis and has been used for alleviating gynecological diseases, including amenorrhea, dysmenorrhea, and paramenia [8]. Wenjing decoction could significantly alleviate pain in women with primary dysmenorrhea and could be used for primary dysmenorrhea treatment [9]. The modified Wenjing decoction repairs abnormal ovarian function in gynecological cold coagulation blood stasis by reducing ovarian oxidative damage [10]. Wenjing decoction is involved in vascular remodeling, microvascular contraction, and microcirculation and helps restore the normal ovarian function in cold coagulation blood stasis syndrome [11]. Therefore, Wenjing decoction alleviates RSA by warming the channel, dispelling cold, nourishing blood, and removing blood stasis. However, the mechanisms underlying Wenjing decoction activity in RSA have not been identified.

TCM typically has multiple components and multiple targets. The active ingredients and candidate targets can be explored by network pharmacology [12, 13]. In this study, the Wenjing decoction was evaluated by active ingredient screening, target protein prediction, cross-validation, enrichment analysis, and protein-protein interaction (PPI) network and pharmacological network construction. Our findings might help understand the pharmacological mechanisms involved in the Wenjing decoction effect in RSA.

## 2. Materials and Methods

### 2.1. Composition of TCM Preparation

The TCM preparation of the Wenjing decoction contained Wuzhuyu (*Tetradium ruticarpum*) 15 g, Danggui (*Angelica sinensis*) 10 g, Chuanxiong (*Ligusticum chuanxiong hort*) 10 g, Guizhi (*Cinnamomum cassia Presl*) 10 g, Shengjiang (*Zingiber officinale Roscoe*) 10 g, Banxia (*Pinellia ternata*) 20 g, Gancao (*Glycyrrhiza uralensis Fisch*) 10 g, Ejiao (*Colla corii asini*) 10 g, Mudanpi (*Paeonia suffruticosa*) 10 g, Chishao (*Radix Paeoniae Rubra*) 10 g, Dangshen (*Codonopsis pilosula*) 10 g, and Maidong (*Ophiopogon japonicus*) 30 g. Based on TCM names, the medicinal ingredient information (including molecular weight (MW), molecule name, lipid-water partition coefficient (AlogP), the number of constituents, the number of hydrogen bond donors/acceptors (Hdon/Hacc), blood-brain barrier (BBB), intestinal epithelial permeability (Caco-2), drug-likeness (DL), oral bioavailability (OB), and drug half-life (HL)) was extracted from the Traditional Chinese Medicine Systems Pharmacology (TCMSP, https://tcmspw.com/index.php) database [14].

### 2.2. Screening of TCM Active Ingredients

The active ingredients were identified using the (ADME) parameters [15] from the TCMSP database. ADME studies evaluate the absorption, distribution, metabolism, and elimination of exogenous chemicals by the body and included DL, HL, and OB. This analysis used conventional screening parameters for drug composition. The cutoff criteria were DL ≥ 0.18 and OB ≥ 30%.

### 2.3. Target Protein Prediction for the Active Ingredients

Using the BATMAN (http://bionet.ncpsb.org/batman-tcm/) [16] and TCMSP [14] databases, the target protein prediction for the active ingredients was performed. The BATMAN database first selected the target proteins from the DrugBank, Therapeutic Target Database (TTD), and Kyoto Encyclopedia of Genes and Genomes (KEGG) databases and ranked the predicted targets in descending order. The target proteins were selected for each small molecule drug candidate using a threshold of a score of >20. The BATMAN database was used to predict the differentiating drug-target interactions using binary classification for target protein prediction of active ingredients. The classification feature eigenvalue of the predicted drug-protein interaction was considered as the maximum eigenvalue of the similarity scores between the predicted drug-protein interaction and all known interactions.

### 2.4. Cross-Validation of the Target Proteins of the Active Ingredients

The raw data from the GSE121950 was downloaded from the Gene Expression Omnibus (GEO, http://www.ncbi.nlm.nih.gov/geo/) database. This dataset was deposited by Huang et al. [17] and included RNA-seq data from decidua tissues on three RSA samples and three control samples. The original reads were filtered as follows: the reads with sequencing adaptor were removed, and the reads with N-base number exceeding one were filtered out. Subsequently, the reads with low-quality (*Q* ≤ 20) bases exceeding 40% of the total bases were deleted, and the reads with length <35 were omitted. Using the Tophat Software (v2.1.0, http://ccb.jhu.edu/software/tophat, the default parameters) [18], the clean reads were mapped to the human reference genome (GRCh38). Based on featureCounts software (v1.6.0, http://subread.sourceforge.net) [19], read count information for the alignment of each gene was obtained following the human gene annotation information provided by GENCODE (Release 25, https://www.gencodegenes.org/) [20]. The reads with the annotation information of “protein_coding” were taken as mRNAs, and the reads with the annotation information of “antisense,” “sense_intronic,” “lincRNA,” “sense_overlapping,” “processed_transcript,” “3 prime_overlapping_ncrna,” and “non_coding” were considered as lncRNAs.

Using the edgeR package (version 3.4, http://www.bioconductor.org/packages/release/bioc/html/edgeR.html) [21], the raw counts were standardized and converted to log2 counts per million (logCPM) values. The differential expression analysis for RSA vs. control groups was conducted. All mRNAs/lncRNAs were analyzed to obtain corresponding *p* values and log fold change (FC) values. The mRNAs/lncRNAs with *p* values of <0.05 and |logFC| > 1 were considered as differentially expressed genes (DEGs). The target protein intersection of the active ingredients and DEGs was analyzed subsequently.

### 2.5. Enrichment Analysis of the Intersected Target Proteins

The Gene Ontology (GO)_biological process (BP) enrichment analysis of the intersected target proteins was conducted using the ClueGO + CluePedia plug-in Cytoscape software (http://www.cytoscape.org) [22]. The adjusted *p* value ≤0.01 was the threshold for GO_BP enrichment analysis. In addition, the R package clusterProfiler [23] was used for the pathway enrichment analysis of the intersected target proteins, and a *p* value ≤0.01 was set as the threshold.

### 2.6. PPI Network for the Intersected Target Proteins and Pharmacological Network Construction

The interactions among the intersected target proteins were predicted by the STRING database (http://string-db.org/) [24], and the PPI network was built using the Cytoscape software [22]. The required confidence (combined score) > 0.4 was the cutoff criterion for the PPI selection. In addition, the pharmacological network involving the TCM-active ingredient-target protein-pathway relationships was built by the Cytoscape software [22].

## 3. Results

### 3.1. Composition of the TCM Preparations and Active Ingredient Screening

The TCM data in the TCMSP database were queried with the keywords to obtain the chemical composition and other relevant information for each TCM. The data showed 176, 125, 189, 220, 265, 116, 280, 55, 119, 134, 55, and 3 constituents in Wuzhuyu, Danggui, Chuanxiong, Guizhi, Shengjiang, Banxia, Gancao, Mudanpi, Chishao, Dangshen, Maidong, and Ejiao, respectively. The constituents were screened further to identify ingredients with the best ADME parameters. There were 31, 2, 7, 7, 5, 13, 93, 11, 29, and 21 active ingredients in Wuzhuyu, Danggui, Chuanxiong, Guizhi, Shengjiang, Banxia, Gancao, Mudanpi, Chishao, and Dangshen, respectively (Table 1).

### 3.2. Target Protein Prediction and Cross-Validation

The BATMAN online tool was used to predict the target proteins for the active ingredients in the TCMSP database, and the target records for 10 herbs, including Wuzhuyu, Danggui, Chuanxiong, Guizhi, Shengjiang, Banxia, Gancao, Mudanpi, Chishao, and Dangshen, were obtained. In total, we identified 933 target proteins of 49 active ingredients from the 10 TCMs after screening the targets with a score larger than 20 in BATMAN.

**Figure 1:**
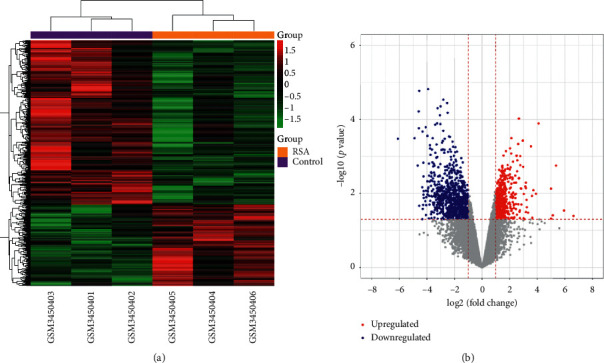
The differential expression analysis of GSE121950. (a) Heatmap displayed the differentially expressed genes (DEGs). Red and green represent up- and downregulation, respectively. Orange and purple represent recurrent spontaneous abortion (RSA) and control groups, respectively. (b) Volcano plot displayed the DEGs. Red and blue dots represent up- and downregulated genes, respectively.

**Figure 2:**
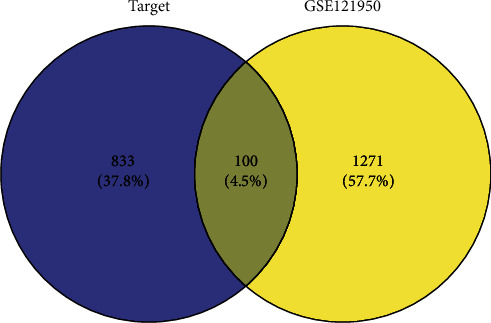
The Venn diagram of the intersected target proteins among the 933 target proteins and 1371 DEGs in GSE121950.

From the raw data of GSE121950, a total of 17163 mRNAs were annotated. Differential expression analysis showed that there were 1371 DEGs (including 453 upregulated and 918 downregulated genes) between RSA and the control samples (Figure 1). The Venn diagram showed the presence of 100 intersecting target proteins among the 933 target proteins of the active ingredients and the 1371 DEGs (Figure 2 and Supplementary Table 1).

### 3.3. Enrichment Analysis of the Intersected Target Proteins

**Figure 3:**
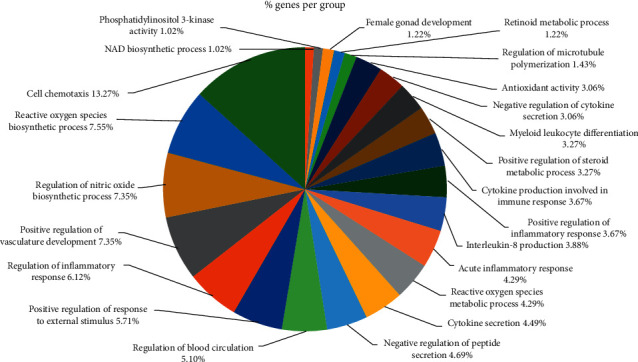
The pie chart showing the proportion of Gene Ontology (GO) terms involving the intersected target proteins.

**Figure 4:**
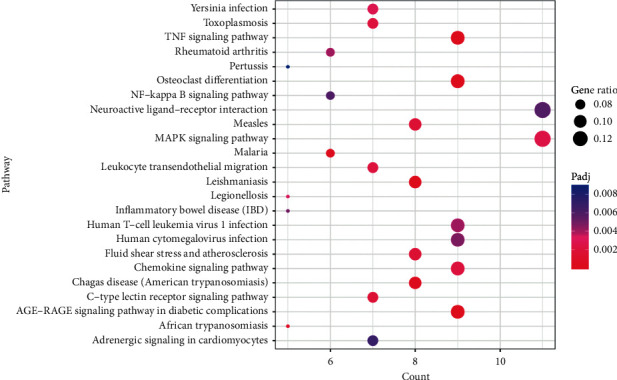
The bubble diagram exhibiting the pathways for the intersected target proteins. The vertical axis represents the pathway name, while the horizontal axis shows the enrichment gene number. The larger the ratio of enriched genes to the total genes, the larger the dot size is. A brighter red dot indicates a smaller *p* value.

For the 100 intersected target proteins, 98 GO_BP terms were enriched significantly. The pie diagram showed the involvement of 23 different functions, such as “cellular chemotaxis” (13.27%), “reactive oxygen species biosynthetic process” (7.55%), “regulation of nitric oxide biosynthesis” (7.35%), “positive regulation of the vasculature development” (7.35%), “regulation of inflammatory response” (6.12%), and others (Figure 3). In addition, 24 KEGG pathways were enriched for the 100 intersected target proteins. The enriched pathways included primarily “AGE-RAGE signaling pathway in diabetic complications,” “TNF signaling pathway,” “osteoclast differentiation,” “C-type lectin receptor signaling pathway,” “chemokine signaling pathway,” “leukocyte transendothelial migration,” and “MAPK signaling pathway”(Figure 4).

### 3.4. PPI Network for the Intersected Target Proteins and Pharmacological Network Construction

**Figure 5:**
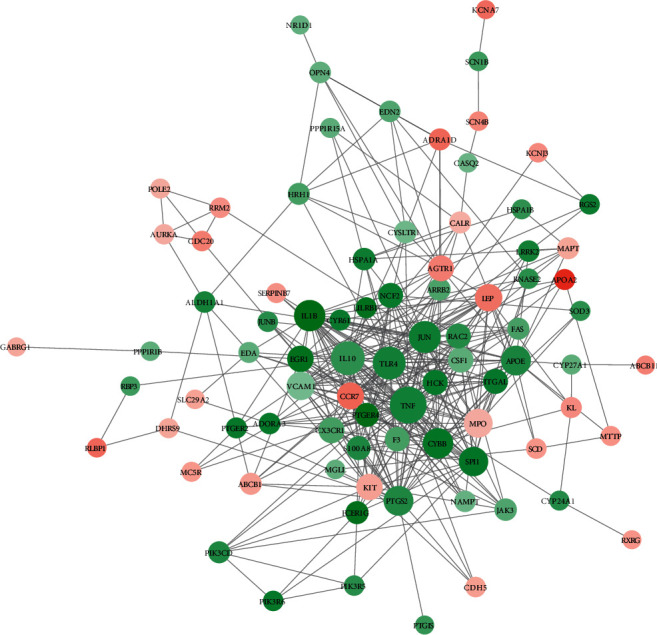
The protein-protein interaction (PPI) network for the intersected target proteins. The stronger the connection, the larger the dot is. Red and green represent up- and downregulation, respectively. Darker color indicates greater fold change.

**Figure 6:**
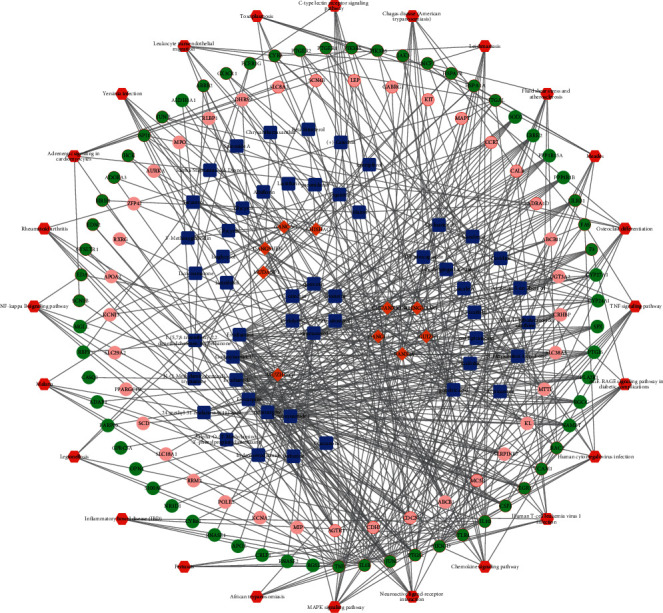
The pharmacological network. Brown diamond, blue square, red and green circles, circles with a red border, and red hexagon represent TCMs, active ingredients, target proteins, pathway-associated target proteins, and pathways, respectively.

In the PPI network for the intersected target proteins, 84 nodes and 365 edges existed (Figure 5). In particular, the top 10 nodes, including tumor necrosis factor-*α* (TNF-*α*), interleukin-10 (IL-10), Toll-like receptor 4 (TLR4), c-Jun (JUN), interleukin-1*β* (IL-1B), cytochrome b558 heavy chain gene (CYBB), prostaglandin-endoperoxide synthase 2 (PTGS2), apolipoprotein E (APOE), Salmonella pathogenicity island 1 (SPI1), and myeloperoxidase (MPO), from the PPI network were screened (Table 2). Combined with the TCM-active ingredient-target protein-pathway relationships, a pharmacological network, involving 10 TCMs, 49 active ingredients, 100 target proteins, and 24 pathways, was constructed (Figure 6).

## 4. Discussion

In this study, we screened 31, 2, 7, 7, 5, 13, 93, 11, 29, and 21 distinct active ingredients from the TCM preparations of Wuzhuyu, Danggui, Chuanxiong, Guizhi, Shengjiang, Banxia, Gancao, Mudanpi, Chishao, and Dangshen. In total, 933 target proteins for the active ingredients in the TCMSP database were predicted and 1371 DEGs between RSA and control samples were identified from GSE121950. Venn diagram showed 100 intersected target proteins between the 933 target proteins of the active ingredients and 1371 DEGs. Among the 100 intersected target proteins, 98 GO_BP terms and 24 pathways were enriched significantly.

The PPI network of the intersected target proteins, TNF-*α*, IL-10, TLR4, JUN, IL-1B, CYBB, PTGS2, APOE, SPI1, and MPO, showed higher degrees. The etiologies of RSA have been extensively identified previously, including chromosomal abnormalities, cervical insufficiency, immune dysfunction, maternal endocrine abnormalities, maternal reproductive tract abnormalities, reproductive tract infection, and thrombotic susceptibility [4, 5]. TNF-*α* overexpression in the decidual tissues and peripheral blood of RSA patients was detected, showing correlation with disease occurrence, and might serve as a candidate indicator for RSA [25, 26]. The *IL-10* levels were significantly lower in RSA than during normal pregnancy, and decreased *IL-10* levels could be correlated to pathologic pregnancies, while increased *IL-10* to healthy pregnancies [27]. The involvement of *TLR4* and C-C chemokine ligand 2 (*CCL2*) in RSA development was shown before, and the serum *TLR4* and *CCL2* levels in pregnant women could serve as RSA indicators [28]. Reduced *TLR4* and increased *TLR2* induced by ligand treatment of spermatozoa influenced unexplained RSA [29]. Therefore, in RSA, *TNF-α*, *IL-10*, and *TLR4* might be targets for Wenjing decoction.

The *IL-1B* (C-511T) polymorphism may promote the development of recurrent miscarriages, while the *IL-6* (G-634C) polymorphism may reduce its risk [30]. Danggui-Honghua by modulating 42 targets, including *PTGS2*, might play a pharmacological role in treating blood stasis syndrome [31]. Apo E3/4, E4/4 genotypes are more frequent in women with recurrent pregnancy loss (RPL) compared to control women, and these genotypes might cause RPL by increasing the thrombophilic risk factors [32]. The levels of the oxidative stress biomarkers, including *MPO*, are higher in women with RPL exhibiting metabolic syndrome, and the oxidative stress, subclinical inflammatory state, and metabolic syndrome contribute to pregnancy outcome prediction in women with RPL [33], thus suggesting that in RSA, *IL-1B*, *PTGS2*, *APOE*, and *MPO* might also be targeted by Wenjing decoction.

Enrichment analysis of the 100 intersected target proteins showed enrichment of “AGE-RAGE signaling pathway in diabetic complications,” “TNF signaling pathway,” “osteoclast differentiation,” “C-type lectin receptor signaling pathway,” “chemokine signaling pathway,” “leukocyte transendothelial migration,” and “MAPK signaling pathway.” A previous study showed the contribution of the C-type lectin receptors in tailoring the adaptive responses and innate recognition, and several C-type lectin receptors function in protective antifungal immunity [34]. Systemic immune responses and abnormal maternal local have shown correlations with RSA; especially, antiphospholipid antibodies and maternal-fetal immune tolerance disturbance affect both autoimmune RSA and alloimmune RSA [35, 36]. Chemokines are a family of chemoattractant proteins, which can trigger immune and inflammatory responses, cell polarization, and prevention of HIV-1 infection [37]. The migration of leukocytes through activated venular walls is a basic immune response, which is necessary for the entry of effector cells into the inflamed tissues [38]. Investigations on the role of AGEs and the receptor of AGEs (RAGE) in vascular disease and blood pressure showed the importance of the AGE-RAGE axis in vascular disease, micro- and macrocirculation [39]. Thus, these pathways could be involved in the pharmacological activity of the Wenjing decoction against RSA. TNF-*α*, IL-10, TLR4, JUN, IL-1B, CYBB, PTGS2, APOE, SPI1, and MPO were involved in the pharmacological network pathways and might be responsible for the pharmacological activity of Wenjing decoction.

This study is a preliminary research to screen the primary targets of Wenjing decoction. RSA is a serious disease involving fetus/baby and mother. Misinformation about RSA may lead to more RSA. Therefore, the results of this study should be interpreted cautiously. Further clinical studies as well as *in vitro* or *in vivo* studies are still warranted to investigate the effects of Wenjing decoction on RSA.

## 5. Conclusions

We selected 100 intersected target proteins of the active ingredients by cross-validation. During RSA, the *TNF-α*, *IL-10*, *TLR4*, *JUN*, *IL-1B*, *CYBB*, *PTGS2*, *APOE*, *SPI1*, and *MPO* pathways might be targeted by the Wenjing decoction. Furthermore, “C-type lectin receptor signaling pathway,” “chemokine signaling pathway,” “leukocyte transendothelial migration,” “TNF signaling pathway,” “MAPK signaling pathway,” and “AGE-RAGE signaling pathway in diabetic complications” might correlate with the pharmacological activity of Wenjing decoction. However, the genes and pathways involved and their significance in women with RSA treated by Wenjing decoction should be explored further.

## Figures and Tables

**Table 1:** The constituents and active ingredients in the traditional Chinese medicines.

Medicine	Before screening (number)	After screening (number)	Source
Wuzhuyu	176	31	TCMSP
Danggui	125	2	TCMSP
Chuanxiong	189	7	TCMSP
Guizhi	220	7	TCMSP
Shengjiang	265	5	TCMSP
Banxia	116	13	TCMSP
Gancao	280	93	TCMSP
Mudanpi	55	11	TCMSP
Chishao	119	29	TCMSP
Dangshen	134	21	TCMSP
Ejiao	3	0	TCMID
Maidong	55	0	TCMID
Total (including the repeats)	1679	219	

TCMSP, Traditional Chinese Medicine Systems Pharmacology; TCMID, Traditional Chinese Medicine Integrative Database.

**Table 2:** The top 10 nodes in the protein-protein interaction network.

Gene	Degree	Betweenness	Closeness
TNF-*α*	37.0	1138.1646	0.58450705
IL-10	31.0	767.9433	0.557047
TLR4	29.0	448.88934	0.5354839
JUN	27.0	832.55804	0.5092025
IL-1B	26.0	255.20244	0.5253165
CYBB	24.0	324.6068	0.51552796
PTGS2	23.0	619.34546	0.5092025
APOE	23.0	805.83795	0.5092025
SPI1	22.0	391.1927	0.47428572
MPO	21.0	217.95062	0.48255813

## Data Availability

The data supporting the conclusions of the study could be accessed by reasonable request from the corresponding author.

## Abbreviation

RSA: Recurrent spontaneous abortion
DEGs: Differentially expressed genes
PPI: Protein-protein interaction
TNF-*α*: Tumor necrosis factor-*α*
IL-10: Interleukin-10
TLR4: Toll-like receptor 4
JUN: Jun proto-oncogene
IL-1B: Interleukin-1-beta
CYBB: Cytochrome b-245 beta chain
PTGS2: Prostaglandin-endoperoxide synthase 2
APOE: Apolipoprotein E
SPI1: Salmonella pathogenicity island 1
MPO: Myeloperoxidase
TCM: Traditional Chinese medicine
MW: Molecular weight
Hdon/Hacc: The number of hydrogen bond donors/acceptors
BBB: Blood-brain barrier
DL: Drug-likeness
OB: Oral bioavailability
HL: Drug half-life
ADME: Absorption, distribution, metabolism, and elimination
TTD: Therapeutic Target Database
KEGG: Kyoto Encyclopedia of Genes and Genomes
GEO: Gene Expression Omnibus
CPM: Counts per million
FC: Fold change
GO: Gene Ontology
BP: Biological process.

## Additional Points

(1) In total, 100 intersecting target proteins were selected by the Venn diagram. (2) Enrichment analysis highlighted 24 pathways for the intersecting target proteins. (3) The key targets include *TNF-α*, *IL-10*, *TLR4*, *JUN*, *IL-1B*, *CYBB*, *PTGS2*, *APOE*, *SPI1*, and *MPO*.

## Supplementary Materials

Supplementary Materials The list of the 100 intersection genes.
