# Supplementary material for: The Pharmacological Activity of the Wenjing Decoction in Recurrent Spontaneous Abortion
Source: Evid Based Complement Alternat Med. 2021 Apr 13;2021:8861394. doi: 10.1155/2021/8861394 (PMC8060116; doi:10.1155/2021/8861394)
Supplement: Supplementary Materials — The list of the 100 intersection genes. [file 8861394.f1.docx]

Supplementary Table 1 The list of the 100 intersection genes

| ID | Gene | logFC | AveExpr | t | P.Value | adj.P.Val | B |
| --- | --- | --- | --- | --- | --- | --- | --- |
| 19 | IL1B | -4.084749 | 1.357494 | -7.671359 | 0.0001625 | 0.1855837 | 1.0054195 |
| 35 | PTGER4 | -3.846655 | -0.155667 | -5.176071 | 0.0015549 | 0.3627859 | -0.69795 |
| 70 | RGCC | -3.485637 | 4.443807 | -4.660467 | 0.0027196 | 0.3654107 | -1.169266 |
| 74 | GPRC5A | -3.446541 | 3.9253064 | -5.051028 | 0.0017747 | 0.3627859 | -0.807928 |
| 80 | RNASE1 | -3.41727 | 6.144809 | -2.802685 | 0.0281559 | 0.4588031 | -3.266442 |
| 84 | FCER1G | -3.379054 | 4.0356425 | -2.888849 | 0.0249909 | 0.4408202 | -3.156854 |
| 86 | EGR1 | -3.370445 | 4.2802064 | -3.97176 | 0.0060928 | 0.3945559 | -1.874849 |
| 92 | LILRB1 | -3.307133 | 0.9967377 | -3.270009 | 0.014916 | 0.4035583 | -2.683713 |
| 139 | CYBB | -3.076809 | 4.7089194 | -3.018036 | 0.0209352 | 0.4183733 | -2.994223 |
| 156 | SPI1 | -3.015078 | 3.416273 | -2.689416 | 0.032977 | 0.4722922 | -3.411682 |
| 169 | HCK | -2.937884 | 3.0775845 | -2.417852 | 0.0484187 | 0.5298938 | -3.763852 |
| 175 | ADORA3 | -2.919854 | -0.672765 | -3.951131 | 0.0062486 | 0.3945559 | -1.897353 |
| 181 | PIK3R6 | -2.890742 | 1.5218704 | -3.488565 | 0.0111975 | 0.3945559 | -2.4225 |
| 189 | ITGAL | -2.859988 | 2.9157567 | -3.241983 | 0.0154824 | 0.4035583 | -2.717776 |
| 194 | PTGER2 | -2.840398 | 1.4255942 | -6.641905 | 0.0003811 | 0.233592 | 0.4049355 |
| 200 | NCF2 | -2.82192 | 3.5148193 | -2.685382 | 0.033164 | 0.4723597 | -3.416876 |
| 207 | CYR61 | -2.804883 | 7.1320767 | -3.527305 | 0.0106504 | 0.3945559 | -2.377044 |
| 224 | RGS2 | -2.723322 | 5.2044353 | -6.026399 | 0.0006671 | 0.2680031 | -0.01872 |
| 227 | JUN | -2.704582 | 6.2002311 | -6.791355 | 0.0003346 | 0.2209049 | 0.5000553 |
| 230 | HSPA1A | -2.687151 | 6.5851767 | -4.987597 | 0.0018993 | 0.363297 | -0.864761 |
| 245 | LRRK2 | -2.593013 | 0.9256346 | -3.219932 | 0.0159443 | 0.4035583 | -2.744662 |
| 250 | TNF | -2.579579 | -0.079048 | -3.566042 | 0.0101324 | 0.3945559 | -2.331855 |
| 254 | TLR4 | -2.562849 | 2.7517719 | -3.465472 | 0.0115381 | 0.3945559 | -2.44972 |
| 264 | ALDH1A1 | -2.527279 | 3.3232568 | -2.549269 | 0.0401734 | 0.504652 | -3.592889 |
| 269 | SPX | -2.519088 | -1.21276 | -4.11631 | 0.0051137 | 0.3929296 | -1.719393 |
| 275 | CRLF1 | -2.499629 | 1.4415511 | -3.386634 | 0.0127886 | 0.3945559 | -2.543334 |
| 291 | APOE | -2.425388 | 7.1126682 | -3.739855 | 0.0081236 | 0.3945559 | -2.132385 |
| 312 | PIK3CD | -2.374589 | 3.0189584 | -4.667071 | 0.0026996 | 0.3654107 | -1.162927 |
| 315 | S100A8 | -2.356131 | 2.003709 | -4.428853 | 0.0035392 | 0.3772616 | -1.396671 |
| 320 | PTGS2 | -2.346398 | -0.812593 | -4.349379 | 0.003881 | 0.3772616 | -1.476998 |
| 345 | RAC2 | -2.289193 | 3.5749808 | -2.789989 | 0.0286571 | 0.4588031 | -3.282659 |
| 354 | CYP24A1 | -2.258987 | 2.099485 | -5.578296 | 0.0010304 | 0.3023044 | -0.36217 |
| 375 | JUNB | -2.207514 | 6.5391477 | -4.384156 | 0.0037272 | 0.3772616 | -1.441703 |
| 379 | PIK3R5 | -2.187266 | 2.0071641 | -3.264855 | 0.0150184 | 0.4035583 | -2.689968 |
| 391 | SOD3 | -2.159271 | 5.8863146 | -2.984369 | 0.0219195 | 0.4289668 | -3.0364 |
| 400 | RNASE2 | -2.136388 | -2.054856 | -2.926554 | 0.0237269 | 0.4382167 | -3.10917 |
| 415 | IL10 | -2.100109 | -1.416275 | -3.981633 | 0.0060197 | 0.3945559 | -1.864107 |
| 427 | HSPA1B | -2.060947 | 7.4220833 | -3.220926 | 0.0159232 | 0.4035583 | -2.743448 |
| 462 | PTGIS | -1.970276 | 2.5183002 | -2.759847 | 0.0298853 | 0.4588031 | -3.321225 |
| 479 | ADAP1 | -1.932275 | -0.152451 | -2.690971 | 0.0329052 | 0.4722922 | -3.40968 |
| 525 | SCN1B | -1.841651 | 1.6801825 | -2.735656 | 0.0309112 | 0.464788 | -3.352242 |
| 538 | RBP3 | -1.816308 | 0.1063644 | -5.90759 | 0.0007469 | 0.2763589 | -0.106771 |
| 565 | CX3CR1 | -1.724901 | 0.6162819 | -3.898604 | 0.0066656 | 0.3945559 | -1.955014 |
| 568 | F3 | -1.723928 | 6.0769138 | -4.652204 | 0.0027449 | 0.3654107 | -1.177209 |
| 571 | HRH1 | -1.719964 | 2.3089122 | -3.441081 | 0.0119102 | 0.3945559 | -2.47857 |
| 596 | FAS | -1.665354 | 2.1026539 | -3.819623 | 0.0073515 | 0.3945559 | -2.042679 |
| 625 | ARRB2 | -1.598116 | 4.6134907 | -3.032603 | 0.0205241 | 0.4183733 | -2.976022 |
| 647 | JAK3 | -1.551916 | 3.9283574 | -2.913664 | 0.0241512 | 0.4395617 | -3.125453 |
| 657 | EDN2 | -1.527714 | -3.305843 | -2.742066 | 0.0306358 | 0.4636702 | -3.344018 |
| 744 | CYP27A1 | -1.356221 | 2.7036913 | -2.645507 | 0.0350736 | 0.4828782 | -3.468297 |
| 746 | CSF1 | -1.354153 | 5.0246883 | -3.160548 | 0.017264 | 0.4150742 | -2.817443 |
| 748 | PLA2R1 | -1.349621 | 3.8881532 | -3.721708 | 0.0083114 | 0.3945559 | -2.152956 |
| 755 | EDA | -1.339357 | 0.4749934 | -2.935911 | 0.0234239 | 0.4362743 | -3.097364 |
| 757 | PPP1R15A | -1.337826 | 7.416746 | -2.491263 | 0.0436164 | 0.5187301 | -3.668256 |
| 758 | PPP1R1B | -1.332424 | -2.146409 | -2.600078 | 0.0373907 | 0.4917521 | -3.527031 |
| 765 | OPN4 | -1.325495 | -3.682499 | -2.769116 | 0.0295018 | 0.4588031 | -3.309355 |
| 771 | MGLL | -1.317223 | 5.9993027 | -2.675441 | 0.0336296 | 0.4750491 | -3.429684 |
| 783 | NR1D1 | -1.290741 | 2.0481114 | -2.828669 | 0.0271589 | 0.4531396 | -3.233307 |
| 799 | PARP10 | -1.253921 | 4.4343544 | -2.577902 | 0.0385796 | 0.4989762 | -3.555755 |
| 811 | NAMPT | -1.229736 | 5.3893142 | -3.509735 | 0.0108948 | 0.3945559 | -2.397628 |
| 834 | CASQ2 | -1.193863 | -2.305276 | -2.484262 | 0.0440522 | 0.5197699 | -3.677364 |
| 866 | CYSLTR1 | -1.118987 | 0.9719883 | -3.132383 | 0.0179306 | 0.4154901 | -2.852147 |
| 892 | VCAM1 | -1.061816 | 5.117353 | -3.108067 | 0.0185285 | 0.4164914 | -2.882204 |
| 926 | CALR | 1.0180931 | 10.678723 | 3.6739744 | 0.0088285 | 0.3945559 | -2.20735 |
| 960 | AURKA | 1.0865953 | 4.2517882 | 2.5715382 | 0.038928 | 0.5008072 | -3.564003 |
| 961 | MPO | 1.0873368 | -0.773732 | 2.5372117 | 0.0408649 | 0.504652 | -3.60854 |
| 966 | POLE2 | 1.1051974 | 2.1202604 | 3.6162887 | 0.0095009 | 0.3945559 | -2.273634 |
| 986 | GABRG1 | 1.1442119 | -3.901138 | 2.8768026 | 0.0254096 | 0.4428515 | -3.172124 |
| 995 | DHRS9 | 1.1561 | 4.1250249 | 3.4509141 | 0.0117586 | 0.3945559 | -2.466926 |
| 1017 | MAPT | 1.184681 | 0.6864637 | 3.3800523 | 0.0128995 | 0.3945559 | -2.551196 |
| 1022 | PPARGC1B | 1.1931424 | 2.1868476 | 2.7740779 | 0.0292986 | 0.4588031 | -3.303005 |
| 1036 | SLC29A2 | 1.2155055 | 4.0704814 | 2.897486 | 0.0246951 | 0.4408202 | -3.145916 |
| 1072 | KIT | 1.2927811 | 2.9729226 | 2.4289454 | 0.0476592 | 0.5284072 | -3.749394 |
| 1078 | MIP | 1.3063749 | -3.193568 | 3.5547167 | 0.0102809 | 0.3945559 | -2.345039 |
| 1088 | CDH5 | 1.3216293 | 6.1152121 | 4.06043 | 0.0054699 | 0.3945559 | -1.779024 |
| 1135 | ABCB1 | 1.412592 | 6.6266445 | 2.442721 | 0.0467332 | 0.5252495 | -3.731446 |
| 1143 | ZFP42 | 1.434376 | 3.7336593 | 3.290778 | 0.0145107 | 0.4023377 | -2.658552 |
| 1148 | MC5R | 1.4557748 | -3.421389 | 3.5604695 | 0.0102052 | 0.3945559 | -2.338339 |
| 1161 | RXRG | 1.4850321 | -1.364715 | 2.7091516 | 0.032078 | 0.4675915 | -3.386289 |
| 1190 | SCD | 1.5258291 | 5.8849314 | 3.3600312 | 0.0132432 | 0.3945559 | -2.575158 |
| 1214 | MTTP | 1.5934188 | -3.193394 | 3.1178328 | 0.0182858 | 0.4164914 | -2.870122 |
| 1224 | SERPINB7 | 1.6229019 | 2.3012895 | 2.8301155 | 0.0271045 | 0.4531396 | -3.231464 |
| 1233 | RRM2 | 1.6554404 | 5.1302766 | 3.3700438 | 0.01307 | 0.3945559 | -2.563166 |
| 1256 | UGT3A2 | 1.7246888 | -1.330332 | 2.8923722 | 0.0248698 | 0.4408202 | -3.152391 |
| 1259 | KL | 1.7393354 | 1.4369446 | 5.9400472 | 0.0007241 | 0.2763589 | -0.082506 |
| 1260 | KCNJ3 | 1.7411619 | -3.232739 | 4.4213872 | 0.0035699 | 0.3772616 | -1.404167 |
| 1270 | SCN4B | 1.8472511 | 0.3412486 | 2.8924889 | 0.0248658 | 0.4408202 | -3.152243 |
| 1282 | ABCB11 | 1.9643619 | -2.806249 | 2.613195 | 0.0367056 | 0.4898745 | -3.510057 |
| 1288 | CDC20 | 2.0309061 | 4.6619971 | 6.1272426 | 0.0006069 | 0.2680031 | 0.0543688 |
| 1291 | AGTR1 | 2.0429249 | 4.0422136 | 2.9493903 | 0.0229947 | 0.4354579 | -3.080376 |
| 1304 | SLC8A3 | 2.1589287 | -2.03801 | 2.8189435 | 0.0275276 | 0.4540114 | -3.2457 |
| 1323 | LEP | 2.3881312 | 10.202666 | 3.5196953 | 0.0107555 | 0.3945559 | -2.385953 |
| 1333 | KCNA7 | 2.5501195 | 3.8723332 | 4.7275028 | 0.0025236 | 0.3654107 | -1.105291 |
| 1334 | SLC38A3 | 2.5629105 | -0.006127 | 5.3674423 | 0.0012751 | 0.34195 | -0.534825 |
| 1335 | RLBP1 | 2.6170977 | -0.809307 | 6.4218509 | 0.0004634 | 0.2565456 | 0.2595174 |
| 1338 | SLC18A1 | 2.674961 | -1.098237 | 8.3943126 | 9.43E-05 | 0.1618773 | 1.3588681 |
| 1340 | ADRA1D | 2.7119628 | -1.098684 | 2.7303834 | 0.0311397 | 0.4648486 | -3.359009 |
| 1344 | CCR7 | 2.7624578 | 6.1314721 | 5.0780683 | 0.0017243 | 0.3627859 | -0.783915 |
| 1360 | CRHBP | 3.5404689 | 1.4332366 | 6.2620025 | 0.0005358 | 0.2680031 | 0.1497328 |
| 1369 | APOA2 | 5.3613236 | -0.703053 | 5.0549047 | 0.0017674 | 0.3627859 | -0.804478 |
